# Enhanced Thermal and Dynamic Mechanical Properties of Synthetic/Natural Hybrid Composites with Graphene Nanoplateletes

**DOI:** 10.3390/polym11071085

**Published:** 2019-06-26

**Authors:** Naveen Jesuarockiam, Mohammad Jawaid, Edi Syams Zainudin, Mohamed Thariq Hameed Sultan, Ridwan Yahaya

**Affiliations:** 1 Department of Mechanical and Manufacturing Engineering, Faculty of Engineering, Universiti Putra Malaysia, UPM Serdang 43400, Selangor, Malaysia; 2 Laboratory of Bio composite Technology, Institute of Tropical Forestry and Forest Products (INTROP), Universiti Putra Malaysia, UPM Serdang 43400, Selangor, Malaysia; 3 Department of Aerospace Engineering, Faculty of Engineering, Universiti Putra Malaysia, UPM Serdang 43400, Selangor, Malaysia; 4 Aerospace Malaysia Innovation Centre (944751-A), Prime Minister’s Department, MIGHT Partnership Hub, Jalan Impact, Cyberjaya 63000, Selangor, Malaysia; 5 Science and Technology Research Institute for Defence, Kajang 43000, Selangor, Malaysia

**Keywords:** kevlar, *cocos nucifera* sheath, epoxy, graphene nanoplatelets, thermal stability, visco elastic properties

## Abstract

The aim of the present research work is to enhance the thermal and dynamic mechanical properties of Kevlar/*Cocos nucifera* sheath (CS)/epoxy composites with graphene nano platelets (GNP). Laminates were fabricated through the hand lay-up method followed by hot pressing. GNP at different wt.% (0.25, 0.5, and 0.75) were incorporated with epoxy resin through ultra-sonication. Kevlar/CS composites with different weight ratios (100/0, 75/25, 50/50, 25/75, 0/100) were fabricated while maintaining a fiber/matrix weight ratio at 45/55. Thermal degradation and viscoelastic properties were evaluated using thermogravimetric analysys (TGA), differential scanning calorimetric (DSC) analysis, and a dynamic mechanical analyser (DMA). The obtained results revealed that Kevlar/CS (25/75) hybrid composites at 0.75 wt.% of GNP exhibited similar thermal stability compared to Kevlar/epoxy (100/0) composites at 0 wt.% of GNP. It has been corroborated with DSC observation that GNP act as a thermal barrier. However, DMA results showed that the Kevlar/CS (50/50) hybrid composites at 0.75 wt.% of GNP exhibited almost equal viscoelastic properties compared to Kevlar/epoxy (100/0) composites at 0 wt.% GNP due to effective crosslinking, which improves the stress transfer rate. Hence, this research proved that Kevlar can be efficiently (50%) replaced with CS at an optimal GNP loading for structural applications.

## 1. Introduction

The ever-burgeoning need for humanity to produce lighter, tougher and cost-effective material has led to the development of composites [1]. Ever since its creation, composites have found themselves to be ubiquitous. Fiber (glass, carbon, Kevlar. etc,)-reinforced polymer composites have been predominantly used in aerospace, automobile, defense, construction and in marine fields because of its higher mechanical, thermal, and viscoelastic behavior [2,3,4]. Nonetheless, it is imperative to find a viable and sustainable alternative to synthetic fibers due to eco-legislation and keenness towards eco-friendly materials [5,6,7].

Recent literature has made it evident that natural fibers can act as a promising and sustainable alternative to man-made synthetic fibers [2,8]. Recent studies demonstrated that *Cocos nucifera* sheath (CS, an agro waste) could be a sustainable reinforcement in polymeric composites (epoxy) [9]. The benefits of using natural fibers are low cost, higher specific strength and low density. Most notably, their inherent biodegradability and renewability encourage the researchers to explore the possibilities of using natural fibers in load-bearing composites structures [10]. However, the major limitations of using plant fibers are low thermal stability, higher moisture uptake and poor interfacial interaction with adjacent counterparts [11,12].

Hybrid composites comprise more than two multi-scale fillers (macro, micro and nano) embedded in a single or multiple polymeric matrix [3]. The benefits of using hybrid composites or multi-scale fillers relies on the fact that the merits of one filler can surpass the limitation of another filler [13]. Hybridizing synthetic and plant fibers in a nanofiller modified polymeric matrix has become an interesting research approach to achieve outstanding mechanical, thermal and viscoelastic properties for advanced structural applications. Also, hybridization reduces the utilization of man-made synthetic fibers, which is one of the main root causes for environmental pollution [6]. The most commonly used nano fillers are nano clay, carbon nanotube (CNT), silicon carbide and carbon nano fibers [14]. Among these fillers CNT exhibited higher strength, stiffness, aspect ratio and specific surface area [15]. Besides its advantages, the major impediment of using CNT is higher production cost which affects the mass production of CNT-filled nanocomposites [16]. The graphene nano platelets (GNP) nano filler could efficiently transfer the stress and improves the load-carrying capability due to large specific surface area. Optimal GNP loading in the polymeric composites, enhanced the mechanical, dynamic mechanical thermal and electrical properties of polymer [17]. GNP is widely used as a nano filler in polymeric nano composites for advanced applications such as super capacitors, batteries, lubricants, defense, and sensors [18,19]. Researchers reported that graphene-based nano composites showed higher strength and stiffness compared to clay or carbon filler-based nano composites with lower cost [20,21].

Thermogravimetric analysis (TGA) is extensively utilized to investigate the thermal degradation of polymeric composites, when the composite specimen is subjected to higher thermal loading [22]. The thermal stability of a material describes its ability to resist mechanical deformation at higher temperature [23]. Many researchers investigated the thermal stability of hybrid synthetic/natural fiber reinforced polymeric composites by using thermogravimetric analysis. Jarukumjorn et al. [24] studied the effect of incorporating sisal fiber on the thermal stability of glass fiber-reinforced polymeric composites. The hybrid composites exhibited higher thermal stability compared to sisal fiber-reinforced polymeric composites. However, the thermal stability of hybrid composites were lower compared to glass fiber-based polymeric composites. Similar observations were found while hybridizing glass/bamboo and glass/sugar palm fiber-based composites [25,26]. Rasana et al. [27] enhanced the thermal stability of glass fiber composites using MWCNT (multi-walled carbon nanotubes). Mourad et al. [28] also utilized different nano fillers and improved the thermal stability of Kevlar fiber-reinforced epoxy composites.

Dynamic mechanical analysis (DMA) is the most widely used technique to measure the viscoelastic properties viz., storage modulus (E′), loss modulus (E″) and damping factor (Tanδ) while applying continuous sinusoidal loads [13,29]. Storage modulus (E′) measures the rigidity and stiffness of the polymeric structure. E′ decreases while increasing the temperature due to the mobility of polymeric chain segments [30,31]. Loss modulus (E”) represents the released energy in terms of heat and it is associated with the viscous response of the polymeric structure. Tan*δ* or damping factor is the ratio between E’ and E’’ [32]. Several works in the literature have corroborated that hybridizing man-made synthetic fibers and plant fibers could improve the viscoelastic properties [33]. Devi et al. [34] studied the viscoelastic properties of glass/pine apple leaf fiber/polyester composites and concluded that inclusion of glass fiber enhanced the viscoelastic properties. Similarly, Cheng et al. [35] also achieved higher viscoelastic properties (higher E′, E″ and lower Tan*δ*) while hybridizing coir and glass compared to coir/polyester composites. On the other hand, nano filler plays a vital role in the relaxation of macromolecular polymeric chains [36]. Pedrazzolli et al. [37] found that addition of graphene nanoplatelets in the glass fiber-reinforced polymeric composites improved the storage modulus and loss modulus while the damping factor has decreased.

From the literature analysis, it was observed that researchers devoted their efforts to replace synthetic fibers by natural fibers through hybridization. However, replacing synthetic fiber with natural fiber may affect the thermal and viscoelastic properties of hybrid composites. Hence, improving the thermal stability and viscoelastic properties of hybrid composites with an efficient nano filler is an interesting and effective research approach to produce high-performance composite structures at lower cost. The primary focus of this research work is to study the influence of incorporating different wt.% of GNP on the thermal degradation and dynamic mechanical properties of Kevlar/epoxy, Kevlar/CS/epoxy and CS/epoxy composites. In particular, previous research reported that Kevlar/CS laminates at 75/25 weight ratio in the absence of nano fillers exhibited almost equal thermal stability and viscoelastic properties compared to Kevlar/epoxy laminates [10]. Nonetheless, higher weight percentage of Kevlar (75%), may still cause environmental issues. This research replaced more weight percentage of man-made synthetic Kevlar fiber with an agro waste (CS) through GNP-modified epoxy composites, due to enhanced interfacial adhesion and effective stress transfer to the reinforcement of the composite system.

## 2. Materials and Methods

### 2.1. Materials

**Figure 1:**
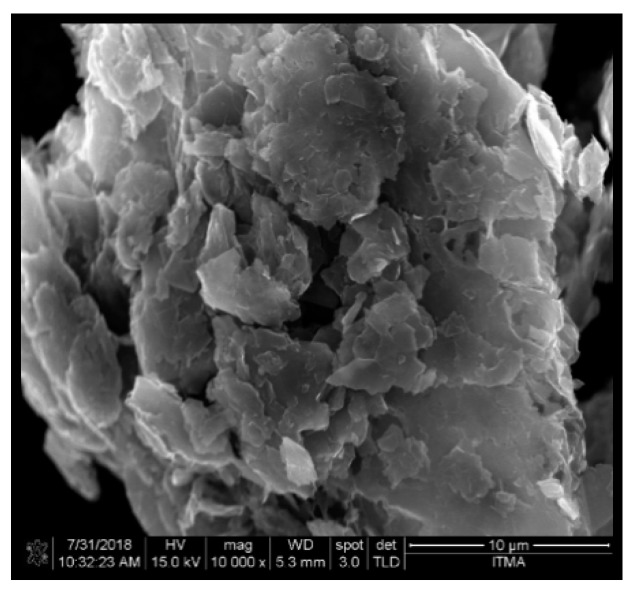
Field-emission scanning electron microscopy (FE–SEM) image of graphene nanoplatelets (GNP).

A “Natural textile” and an “Agro wastes”, so called *Cocos nucifera* sheath (CS) was procured from the villages of Serdang, Malaysia. The density of the CS varied between 1.37–1.50 g/cm^3^. The chemical compositions of CS were as follows: cellulose (21–22%), hemicellulose (42–43%), lignin (31–33%) and extractives (2–2.5%). The para aramid fabric used in this research was Kevlar 29. (Thickness: 0.33 mm, density: 1.44g/cm^3^). D.E.R 331 epoxy resin with joint amine hardener (905-3S) were purchased from T.E.M. engineering bhd. (Puchong, Selangor, Malaysia). Graphene nanoplatelets (GNP) were procured from Sigma Aldrich, St. Louis, MO, USA. Figure 1 displays the field-emission scanning electron microscopy (FE–SEM) image of the GNP. The properties of GNP were as follows: surface area: 110m^2^/g, bulk density: 0.2 g/cm^3^ and lateral dimension: 2–3 μm).

### 2.2. Fabrication of Graphene Nanoplatelets (GNP) Modified Laminated Composites

**Figure 2:**
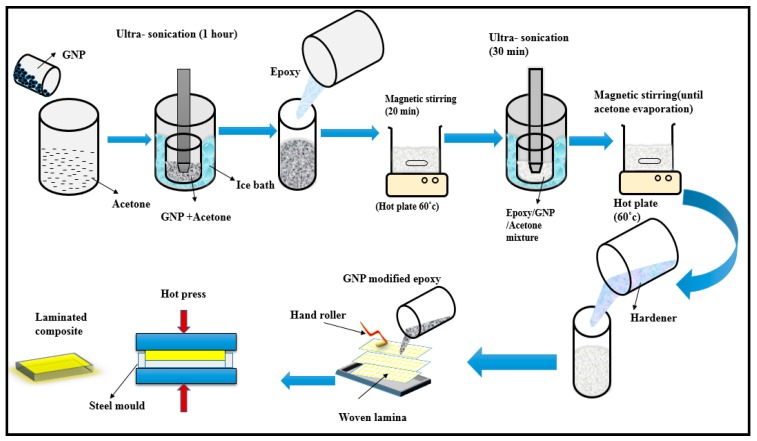
GNP incorporation into epoxy and fabrication of laminates [39].

Figure 2 illustrates the process of GNP inclusion into epoxy. Initially, the required quantity of GNP were pre-dispersed in acetone solution (15 mg/mL) using an ultra-sonicator for 1 h. The GNP/acetone solution was mixed with epoxy using a magnetic stirrer over a hot plate. In order to achieve uniform dispersion, the GNP/epoxy mixture underwent sonication process using an ultrasonicator for 30 min at 100 W. The acetone present in the mixture could be removed when the solution was kept over a hot plate (60 °C) for 20 min with a magnetic stirrer [38]. The GNP/epoxy solution obtained was cooled at room temperature for 1 h. Finally, the joint amine hardener was mixed with GNP/epoxy mixture.

**Figure 3:**
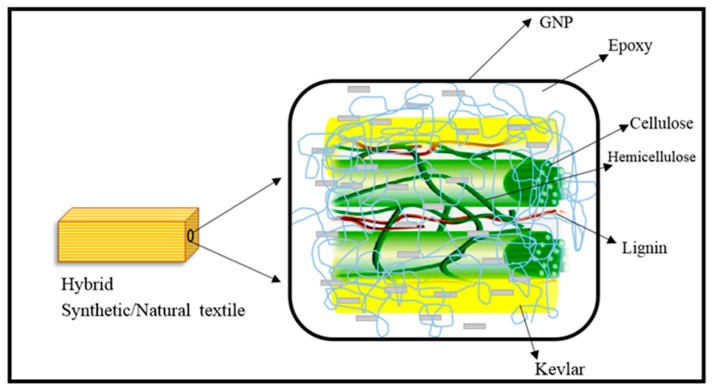
Schematic of different constituents of hybrid composites.

Laminated composite samples were fabricated using the hand lay-up approach followed by hot pressing. A stainless steel mould with a dimensions of 150 × 150 × 3 mm^3^ was employed to fabricate the laminates. Initially, the mould was sprayed with a releasing agent (silicone spray), which improves the finishing and prevents the adhesion with the steel mould. The weight ratio of reinforcement and matrix was maintained to 45/55. On the other hand, the weight ratios of Kevlar/CS were maintained at 100/0 (S1), 75/25 (S2), 50/50 (S3), 25/75 (S4), and 0/100 (S5). Table 1 displays the laminate’s specification, number of layers, layering arrangement and GNP wt.%. The woven Kevlar and CS laminae were positioned inside the mould according to the layering pattern and wetted with GNP-modified epoxy. Hand roller was used to eliminate the air bubbles in between the laminae. Eventually, the samples were cured in hot press at 105 °C for 1 h (Pressure: 275 bar). Figure 3 illustrates the different constituents presents in the hybrid composites. The GNP could form a strong interface between the reinforcement and matrix.

### 2.3. Experimentation 

#### 2.3.1. Thermogravimetric Analysis (TGA)

The influence of adding GNP on the thermal stability of Kevlar/epoxy, CS/epoxy and hybrid Kevlar/CS composites was investigated using a thermo gravimetric analyzer (Mettler Toledo TGA 1, Mettler Toledo, Columbus, OH, USA) as per ASTM E1131-03 standards. As per the standard, the powdered composites (20 mg) were placed in an alumina crucible and underwent pyrolysis process in N_2_ environment (N_2_ flow rate: 50 mL/min). The analysis was carried out from room temperature to 900 °C (heating rate: 10 °C/min).

#### 2.3.2. Differential Scanning Calorimetry (DSC)

Differential scanning calorimetry (DSC) testing was carried out using a DSC analyzer (Mettler Toledo, Mettler Toledo, Columbus, OH, USA). Powdered composite sample of 20 mg was placed inside the crucible pan (aluminum). Another crucible pan was employed as a reference without any sample. The DSC testing has been carried out from room temperature to 900 °C under a nitrogen purge (N_2_ flow rate: 50 mL/min) at a heating rate of 10 °C/min.

#### 2.3.3. Dynamic Mechanical Analysis (DMA)

A dynamic mechanical analyser (TA: DMA Q 800, TA Instruments, New Castle, DE, USA) was employed to evaluate the effect of adding GNP on the viscoelastic properties of different composites laminates. Dynamic mechanical analysis has been conducted in accordance with ASTMD 4065 standards. The dimensions of the test samples were 60 × 12.5 × 3 mm^3^. The laminates have undergone three point bending under a controlled sinusoidal loading condition at 1 Hz frequency. Also, the analysis was performed from 30 °C to 200 °C (heating rate: 10 °C/min).

## 3. Results and Discussion

### 3.1. Thermogravimetric Analysis (TGA)

**Figure 4:**
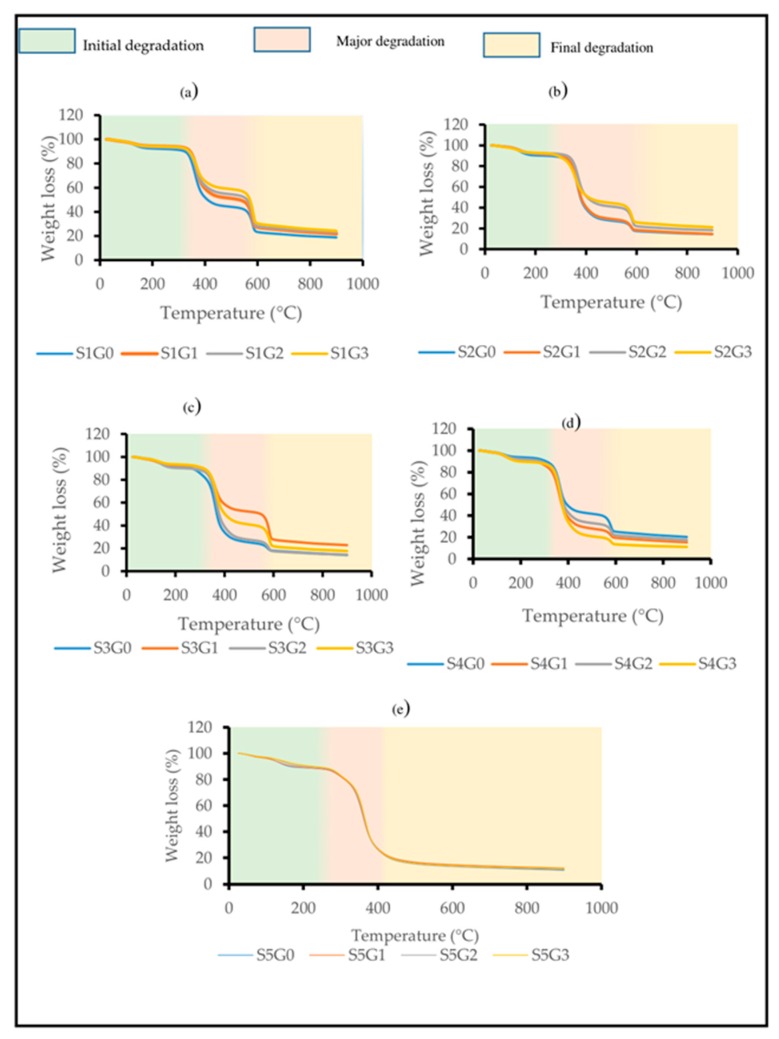
Effect of GNP on the thermal stability of laminated composites. (**a**) S1, (**b**) S2, (**c**) S3, (**d**) S4, (**e**) S5.

TGA analysis has been carried out for Kevlar/epoxy, CS/epoxy and Kevlar/CS hybrid composites at different GNP wt.%. Figure 4 clearly depicts the effect of adding GNP on the thermal stability of Kevlar/epoxy (Figure 4a), hybrid composites (Figure 4b–d) and CS/epoxy composites (Figure 4e). From all the thermograms it was observed that the initial degradation of all the composites samples occur at 100–150 °C due to the elimination of moisture through dehydration of secondary alcoholic groups and forms a unsaturated structure [40]. The unsaturation has led to a weak aliphatic C–O and C–N bonds [41]. The second stage of degradation or the major degradation could be observed above 300 °C because of the decomposition of aromatic epoxy and aliphatic amine hardener. Also, the chemical compositions presents in the CS degraded in the following temperature range, cellulose: 275–500 °C, hemicellulose: 150–350 °C and lignin 250–500 °C [42]. This is mainly attributed to the scission of C–N and C–O which accelerates the volatilization [41]. The third phase of degradation occur beyond 500 °C. This is due to the scission of hydrogen bonds of aromatic polyamide (Kevlar) together with an efficient nano-thermal barrier (GNP). This can be clearly seen from Figure 4a–d. However, due to the absence of aromatic polyamide in CS/epoxy composites, it degraded before 400 °C (Figure 4e).

It has been noticed that the addition of GNP enhanced the thermal stability of all the composite samples. It is well known that Kevlar/epoxy composites possess higher thermal stability while CS/epoxy composites degraded at lower temperature. Thermal stability of Kevlar/epoxy (S1G0, S1G1, S1G2, and S1G3) and CS/epoxy composites (S5G0, S5G1, S5G2, and S5G3) were also examined at different wt.% of GNP to compare the results with hybrid composites. The addition of GNP has shown only slight improvement in thermal stability for Kevlar/epoxy (S1G0, S1G1, S1G2, and S1G3) and CS/epoxy composites (S5G0, S5G1, S5G2, and S5G3). Fan et al. [43] found that the addition of nano filler (MWCNT) in the Kevlar/epoxy composites have shown the decomposition temperature of around 565 ± 10 °C. Whereas, the final decomposition temperature of Kevlar/epoxy/GNP composites in the present research work is 585 °C which proves that addition of GNP exhibited better thermal stability compared to MWCNT.

Nonetheless, hybridizing Kevlar and CS in the GNP modified epoxy composites will combine the properties of different constituents and results in higher thermal stability. Previous research has proven that the thermal stability of Kevlar/CS hybrid composites (75/25) is significantly closer to Kevlar/epoxy composites [10]. However, higher wt.% of Kevlar (75%) in the hybrid Kevlar/CS/epoxy could pollute the environment and affect the eco system. Hence, this research has been dedicated to replace more Kevlar content using an agro waste (CS) without compromising the thermal stability by using GNP.

**Figure 5:**
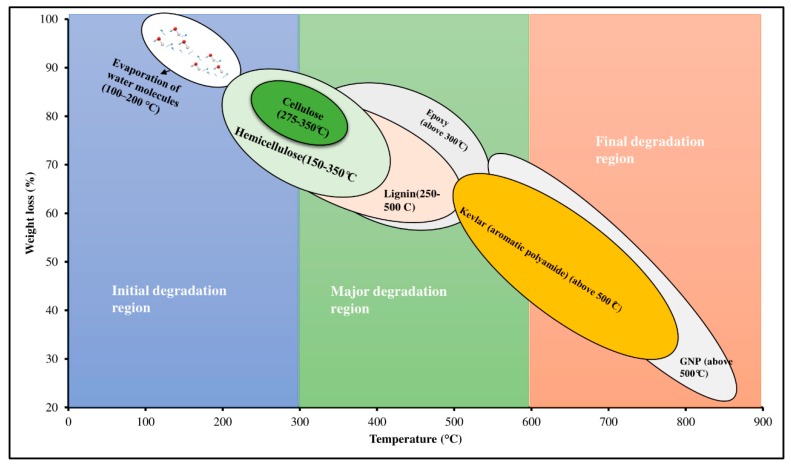
Graphical representation of thermal degradation pathway of Kevlar/*Cocos nucifera* sheath (CS)/GNP epoxy hybrid composite.

From Table 2, it is clear that the addition of GNP improved the thermal stability of all the laminated composites irrespective of individual composition. On the other hand, the hybrid composites S2G3, S3G3 exhibited higher major degradation and final degradation temperature compared to Kevlar/epoxy composites (S1G0). However, thermal stability of S4G3 is almost closer to S1G0. This is mainly attributed to the multi-scale fillers acting as a thermal barrier which delayed the volatilization [44]. From the thermal analysis, it was observed the S1G0 (Kevlar/epoxy) composites could be efficiently replaced with *Cocos nucifera* sheath (75%) at 0.75 wt.% of GNP (S4G3). Moreover, Figure 5 clearly shows the thermal degradation pathway of Kevlar/CS/GNP epoxy composites. From the graphs it can be understood that the GNP delayed the volatilization.

### 3.2. Differential Scanning Calorimetry

**Figure 6:**
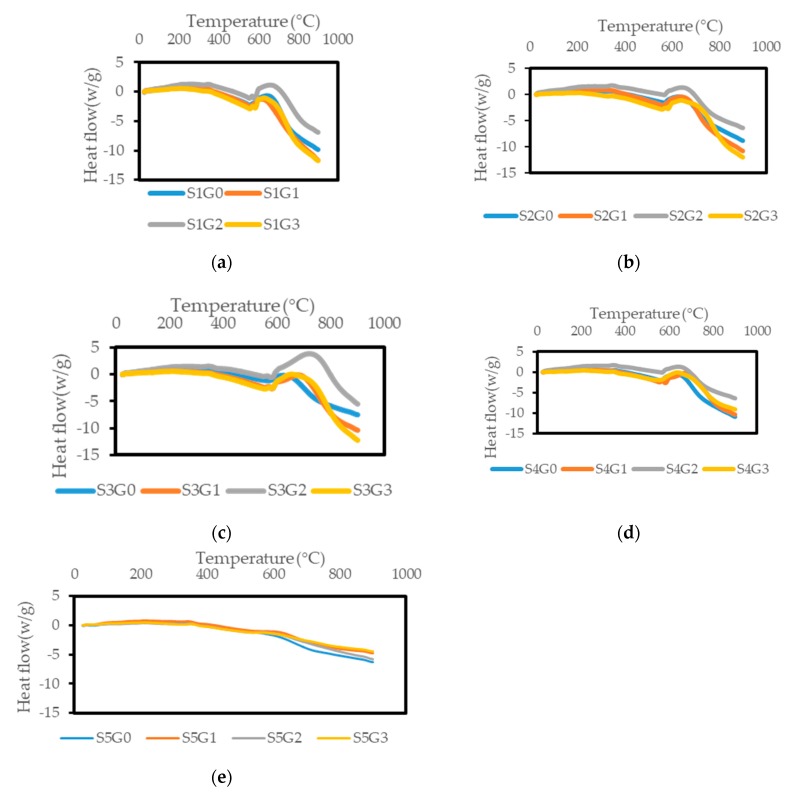
Effect of GNP on the differential scanning calorimetry (DSC) plots of laminated composites. (**a**) S1, (**b**) S2, (**c**) S3, (**d**) S4, (**e**) S5.

Figure 6 displays the effect of adding GNP on the DSC plots of Kevlar/epoxy (Figure 6a), hybrid composites (Figure 6b–d) and CS/epoxy composites (Figure 6e). While performing DSC analysis, the heat flow were measured as a function of time and temperature to investigate the glass transition temperature (T_g_), curing temperature and decomposition temperature [28,45]. The DSC curves of all the composite samples follows a similar pattern after adding GNP. Due to the thermoset polymeric matrix (epoxy), the composites have undergone direct decomposition. As a consequence, it is not possible to find the degree of crystallinity for thermoset polymeric composites [46,47,48]. Nonetheless, T_g_ and decomposition temperature would be investigated through endothermic transition. The upper peak in the DSC curve represents the endothermic process while the lower peak indicates the exothermic process.

From the analysis the initial endothermic peak around 60–70 °C represents the T_g_ of the epoxy [10]. Addition of GNP shifted the T_g_ to higher temperature (70–80 °C) at 0.25 and 0.5 wt.% of GNP. However, at higher GNP wt.% (0.75) there was not much improvement in T_g_ due to the agglomeration at the interface of the polymer and reinforcement which hinders the polymer cross linking [49,50]. No exothermic peak was observed in any case which corroborated the compete cure of all the composites [51]. The next endothermic region found around 350–380 °C was the decomposition of aromatic epoxy and aliphatic amine hardener [41]. The final endothermic transition occur at 380–600 °C (for S1, S2, S3 and S4) which is attributed to the degradation of Kevlar, CS, GNP and epoxy. On the other hand, the decomposition of CS/epoxy composites was observed at 350–400 °C. Similar findings were reported by other researchers on coir/epoxy [41]. The temperature shifts was found to be higher for the composites with GNP. Hence, the addition of GNP at 0.5 wt.% restricts the movement of polymeric chain and thereby improves the decomposition temperature of Kevlar, hybrid Kevlar/CS and CS/epoxy composites. However, at higher GNP wt.% (0.75) there was no change in decomposition temperature. A similar trend was observed by Xia et al. [51] when using GNP filler in epoxy. DSC results were similar to TGA results and revealed that the composite samples (S1, S2, S3, S4, and S5) after adding GNP exhibited virtuous resistance or stability towards heat in the epoxy composites. Also, it was observed the S1G0 composites could be efficiently replaced with CS at 0.75 wt.% of GNP (S4G3).

### 3.3. Dynamic Mechanical Analysis (DMA)

#### 3.3.1. Storage Modulus (E’)

**Figure 7:**
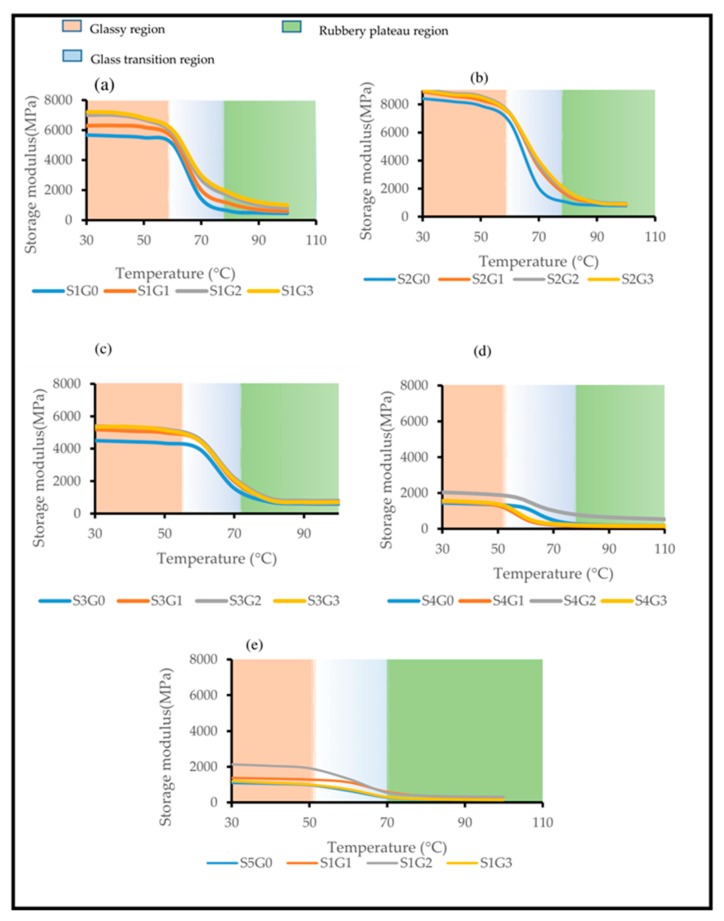
Effect of GNP on the storage modulus of laminated composites. (**a**) S1, (**b**) S2, (**c**) S3, (**d**) S4, (**e**) S5.

Figure 7 displays the impact of adding GNP on E’ of Kevlar/epoxy (Figure 7a), hybrid composites (Figure 7b–d) and CS/epoxy composites (Figure 7e). The stiffness and rigidity of the composite structure were investigated through storage modulus curve [52,53]. The storage modulus plot comprises (Figure 7a–e) of three phases while increasing the temperature. During the first phase the composite structure is very stiff and rigid due to the tightly packed molecules. It is attributed to the rigid polymeric chain. In the second glass transition phase, the storage modulus (E’) was found to be decreased above T_g_ because of the polymeric chain movement. Movement in polymeric chain affects the stiffness as well as the fiber/matrix adhesion [54]. During the third phase (rubbery plateau region), there was no major difference in E’ due to accelerated polymeric chain mobility at higher temperature [30]. From the observation it is clear that the addition of GNP has shown significant change in E’, only in the rigid and T_g_ regions, while in the rubbery region overlapping E’ plots has proved that there was no notable change in E’ due to GNP [29].

Figure 7 a–c clearly displays that, incorporation of GNP at 0.25 wt.% (S1G1, S2G1, and S3G1) and 0.5 wt.% (S1G2, S2G2, S3G2) improved the storage modulus of S1, S2 and S3 laminates.

However, at 0.75 wt.% of GNP (S1G3, S2G3, S3G3) there was not much improvement of storage modulus observed for S1, S2 and S3 laminates. From Figure 7d,e it was found that inclusion of GNP up to 0.5 wt.% (S4G2, S5G2) enhanced the storage modulus due to the reduction in polymeric chain movement because of stiffening effect [27]. Also, it is attributed to the fact that nano filler-modified epoxy matrix improved the stress transfer rate from matrix to the reinforcement [27,36]. Nevertheless, at 0.75 wt.% of GNP (S4G3, S5G3) the storage modulus has declined due to GNP aggregation and stress concentration. This synergistic effect can be seen vividly from S4 and S5 laminates (Figure 7d,e). Similar observations were reported by Rasana et al. [27] that higher nano filler loading (CNT) declined in the storage modulus of glass fiber reinforced epoxy composites.

It is well known that man-made synthetic fiber-based composites possess higher storage modulus compared to natural fiber-based composites [55]. However, hybridizing Kevlar, CS in the GNP modified epoxy could take advantages of individual load carrying constituent and results in superior properties [3]. While comparing all the laminated composites, S2G3 laminates exhibited higher storage modulus [10]. However, S2 laminates contains higher Kevlar content (75%). The aim of this research was replacing S1G0 laminates (contains 100 wt.% of Kevlar) with more *Cocos nucifera* sheath content at an optimal GNP wt.% without affecting performance. From that point of view, S3G3 laminates (contains 50 wt.% CS at 0.75 wt.% of GNP) exhibited almost similar E’ value compared to S1G0. Hence, S3G3 laminates can efficiently replace the Kevlar/epoxy composites (S1G0).

#### 3.3.2. Loss Modulus (E”)

**Figure 8:**
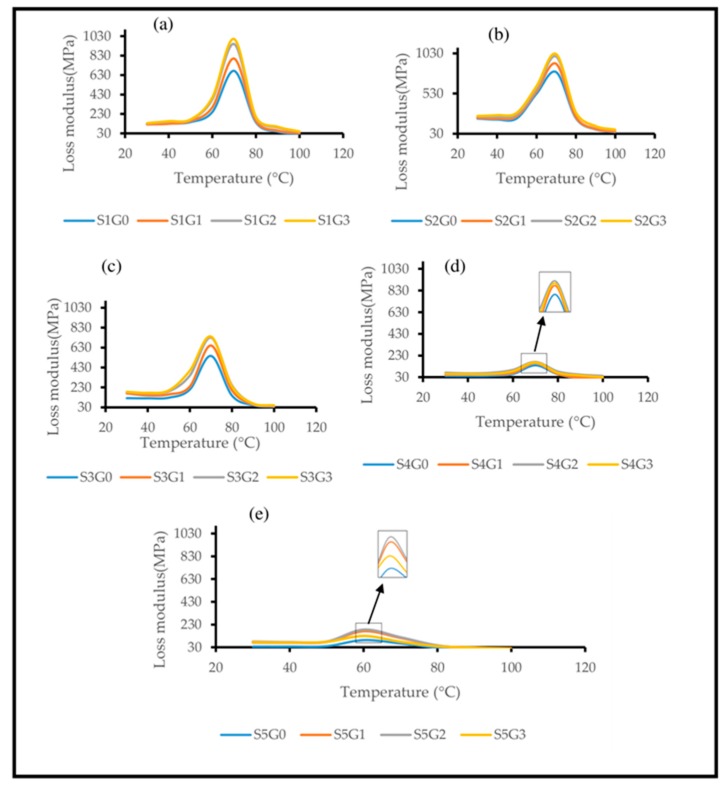
Effect of GNP on the loss modulus of laminated composites. (**a**) S1, (**b**) S2, (**c**) S3, (**d**) S4, (**e**) S5.

Loss modulus is a measure of released heat energy per cycle in a sinusoidal loading condition for a viscoelastic material [29]. Figure 8 delineates the influence of adding GNP on E” of Kevlar/epoxy (Figure 8a), hybrid composites (Figure 8b–d) and CS/epoxy composites (Figure 8e) as a function of temperature. It has been noticed from all the graphs that E” value increases, attained a maximum value and then declined in the T_g_ temperature range for all the laminates irrespective of the individual constituents. Moreover, all the laminates manifested two relaxations localized at T_g_ (65 °C to 75 °C) and 80–90 °C. Nevertheless, at higher temperature, overlapping of curves indicates that inclusion of GNP has no significant effect on the viscous dissipation [29].

Figure 8a–c shows that inclusion of GNP improved the loss modulus of S1G0, S2G0 and S3G0 laminates. In particular, at 0.25 wt.% of GNP the laminates S1G1, S2G1, S3G1 enhanced the loss modulus by 19.04%, 12.91% and 19.26%, respectively. At 0.5 wt.% of GNP the laminates S1G2, S2G2, S3G2 improved the loss modulus by 40.01%, 24.215 and 39.34%, respectively, compared to S1G0, S2G0 and S3G0 laminates, demonstrating improved energy dissipation and higher mechanical properties. However, at 0.75 wt.% of GNP (S1G3, S2G3, and S3G3) there was not much improvement of loss modulus.

Figure 8d,e shows that addition of GNP up to 0.5 wt.% (S4G2, S5G2) enhanced the loss modulus by 21.4% and 98.8 % compared to S4G0 and S5G0 laminates, respectively. This is due to the improved fiber/matix adhesion which hinder the polymeric chain movement and form a rigid polymeric structure [36]. However, at higher GNP concentration (S4G3, S5G3) the loss modulus has been declined because of GNP agglomeration which loosens the polymeric structure. Similar findings were reported by Rasana et al. [27] that the addition of higher nano filler (CNT) decreased the viscous dissipation of glass fiber-reinforced epoxy composites. Hossain et al. [14] also reported that higher GNP loading declined the E” of polymeric composites. Similar to storage modulus results, S2G3 laminates exhibited higher loss modulus [10]. However, S2 laminates contain higher Kevlar content (75%). As per the notion of this research, S3G3 laminates (contains 50 wt.% CS at 0.75 wt.% of GNP) exhibited almost similar E” value compared to S1G0. Hence, S3G3 laminates can act as an alternative to S1G0 composites.

#### 3.3.3. Tan Delta (Tanδ)

**Figure 9:**
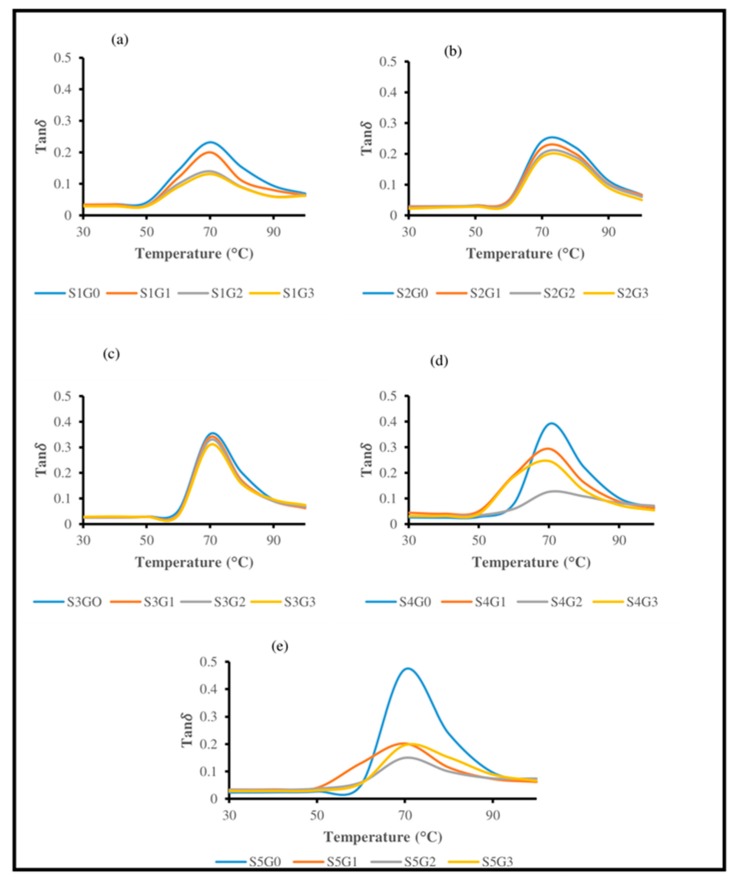
Effect of GNP on Tan ***δ*** plot of laminated composites. (**a**) S1, (**b**) S2, (**c**) S3, (**d**) S4, (**e**) S5.

Figure 9 shows the effect of adding GNP on the Tanδ plots of Kevlar/epoxy (Figure 9a), hybrid composites (Figure 9b–d) and CS/epoxy composites (Figure 9e) as a function of temperature at 1 Hz frequency. Reinforcement and interfacial interactions play a vital role on the damping factor [29]. Glass transition temperature (T_g_) could be obtained from the peak of both loss modulus as well as Tanδ plots. Nevertheless, T_g_ values observed in the loss modulus plot were more reliable compared to T_g_ from the damping plot [56]. T_g_ obtained from E” and Tanδ are shown in Table 3. Similar observations were perceived in the case of hybrid glass/sugar palm and jute/oil palm composites [1,33]. Lower damping values in Tanδ plots represents the improved interfacial interactions, while higher Tanδ value at T_g_ indicates the lack of interfacial adhesion [10].

Addition of GNP at 0.25 wt.% (S1G1, S2G1, and S3G1) and 0.5 wt.% (S1G2, S2G2, S3G2) drastically declined the Tanδ due to enhanced interfacial interactions (Figure 9a–c). Idicula et al. [57] reported that the composites with lower Tanδ associated with T_g_ could withstand higher load. This clearly indicates the improved load carrying capability of the composites after adding GNP. This is mainly attributed to the restriction in intermolecular movement of the polymeric chain [14,27].

However, at higher GNP loading (S1G3, S2G3, and S3G3) minimal deviation was observed compared to the composites with 0.5 wt.% of GNP (S1G2, S2G2, S3G2). Figure 9d,e shows that addition of GNP up to 0.5 wt.% (S4G2, S5G2 ) declined the Tanδ due to uniform GNP dispersion, which makes the polymeric structure more stiff and rigid because of the hindrance in polymeric chain mobility [36]. Nonetheless, the composites with higher GNP concentration (S4G3, S5G3) have shown poor damping behaviour (higher Tanδ) due to the agglomeration of nano fillers together with higher stress concentration in the vicinity of the interphase. Hossain et al. [14] also reported that higher GNP loading exhibited higher Tanδ due to the polymeric chain movement and reduction in load-carrying capability. Table 3 shows the peak height of damping curve which further corroborates the storage modulus and loss modulus results.

#### 3.3.4. Cole–Cole Plot

**Figure 10:**
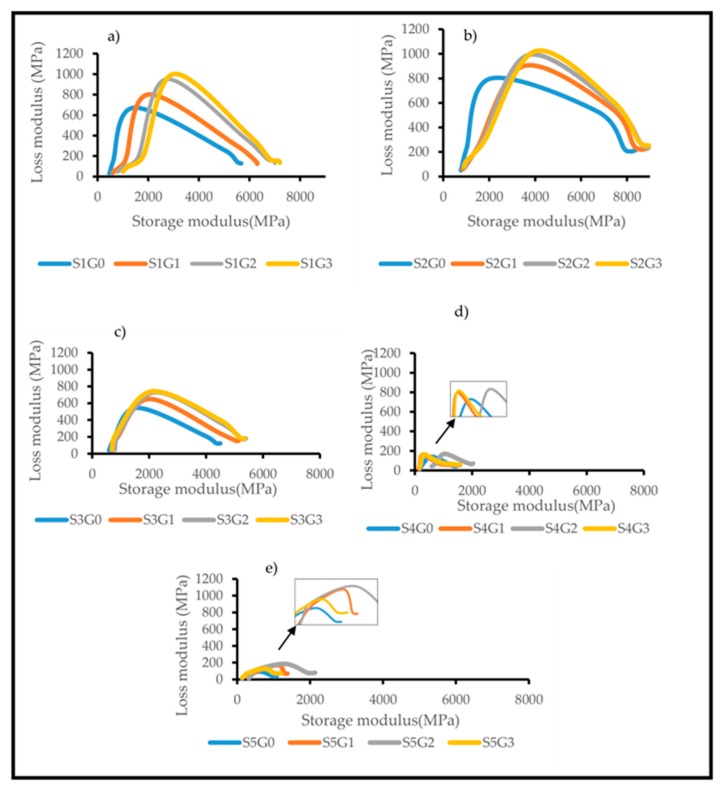
Effect of GNP on the Cole–Cole plot of laminated composites. (**a**) S1, (**b**) S2, (**c**) S3, (**d**) S4, (**e**) S5.

The Cole–Cole plot is a viable tool to interpret the relation between stored and dissipated heat energy for a viscoelastic material [30]. Moreover, it is predominantly used to study the change in polymeric structure after incorporating different micro, macro and nano reinforcements. Cole–Cole plots were constructed with E” of different composite samples as a function of E’ [1]. Figure 10 shows the influence of adding GNP on the Cole–Cole plots of Kevlar/epoxy (Figure 9a), hybrid composites (Figure 10b–d) and CS/epoxy composites (Figure 10e). The homogeneous or heterogeneous nature of polymeric structure could be identified from the shape of the Cole–Cole plots [34]. A perfect semicircular arc in the Cole–Cole plots represents the homogeneity of the system. On the other hand, an irregular or imperfect semicircular curve is an indication of heterogeneous structure [32].

From Figure 10a–d, it has been understood that all the curves follow an imperfect semi-circular shape which corroborates the heterogeneous nature of the composites. However, Figure 10a–c clearly depicts the fact that inclusion of GNP reduces the imperfectness of (S1G1, S1G2, S1G3, S2G1, S2G2 and S2G3) the Cole–Cole plot compared to the composites without GNP (S1G0, S2G0 and S3G0). It is attributed to the enhanced interfacial interactions with Kevlar/CS and epoxy through GNP. Figure 10d,e also shows that the addition of GNP decreased the imperfectness of the Cole–Cole plot. Remarkably, at higher GNP (S4G3 and S5G3) the curve follows an imperfect semi-circular shape due to GNP agglomeration. From the observation, it has been noticed that addition of GNP slightly enhanced the homogeneity of Kevlar, hybrid and CS-based epoxy composites. Moreover, the type of reinforcement and the weight % plays a vital role in the Cole–Cole plots, thereby affecting the viscoelastic behaviour of Kevlar, CS and hybrid Kevlar/CS composites.

## 4. Conclusions 

The effects of adding GNP on the thermal stability and viscoelastic properties of Kevlar/epoxy, CS/epoxy and hybrid composites were evaluated as a function of temperature. The TGA results obtained confirmed that thermal degradation of S4G3 (hybrid composites) is almost closer to S1G0 (Kevlar/epoxy) due to GNP, which act as a thermal barrier and delayed the volatilization. From the dynamic mechanical analysis, it was observed that the storage modulus and loss modulus of S3G3 laminates (contains 50/50 weight ratio of Kevlar/ CS at 0.75 wt.% of GNP) exhibited almost similar E’ and E” value compared to S1G0 (Kevlar/epoxy). Also, S3G3 laminates exhibited higher compatibility between the multi-scale filler and epoxy with lower Tanδ. Moreover, S3G3 laminates exhibited rigid polymeric structure due to the restriction in intermolecular movement which enhanced the stress transfer rate and load-carrying capability. Overall, from the thermogravimetic and dynamic mechanical analysis it has been concluded that the hybrid composites S4G3 and S3G3 could efficiently replace Kevlar/epoxy (S1G0) polymeric structure at elevated temperature.

## Figures and Tables

**Table 1:** Specification and its corresponding layering sequence and GNP wt.%.

Sl.No	Symbol	No of Layers		Layering Sequence	GNP (wt.%)
		Kevlar	CS		
1.	S1G0	4	0	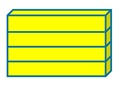	0
2.	S1G1	4	0		0.25
3.	S1G2	4	0		0.50
4.	S1G3	4	0		0.75
5.	S2G0	3	1	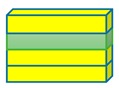	0
6.	S2G1	3	1		0.25
7.	S2G2	3	1		0.50
8.	S2G3	3	1		0.75
9.	S3G0	2	2	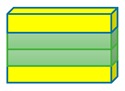	0
10.	S3G1	2	2		0.25
11.	S3G2	2	2		0.50
12.	S3G3	2	2		0.75
13.	S4G0	1	3	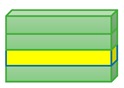	0
14.	S4G1	1	3		0.25
15.	S4G2	1	3		0.50
16.	S4G3	1	3		0.75
17.	S5G0	0	4	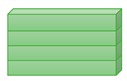	0
18.	S5G1	0	4		0.25
19.	S5G2	0	4		0.50
20.	S5G3	0	4		0.75

Kevlar 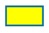, CS 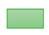.

**Table 2:** Thermal degradation and char residue of different wt.% of Kevlar/CNS composites.

Laminated Composites	Major Degradation Temperature (°C)	Weight Loss (%)	Final Degradation Temp. (°C)	Char Residue (%)
S1G0	371	42.65	582	21.83
S1G1	372	40.68	583	22.01
S1G2	372	38.87	584	23.32
S1G3	374	34.92	585	24.28
S2G0	369	62.62	581	14.67
S2G1	370	47.99	582	21.17
S2G2	370	62.21	582	22.10
S2G3	371	51.11	583	22.78
S3GO	366	66.76	579	14.19
S3G1	367	40.33	583	16.71
S3G2	369	46.62	583	19.86
S3G3	371	52.37	583	22.87
S4G0	365	68.79	570	12.23
S4G1	366	63.10	581	15.36
S4G2	367	58.98	582	16.50
S4G3	369	69.01	582	17.75
S5G0	363	73.82	390	11.12
S5G1	364	73.57	391	12.22
S5G2	365	75.96	391	11.81
S5G3	366	66.96	391	11.76

**Table 3:** Glass transition temperature (T_g_) and Tanδ peak of laminated composites.

Laminates	Tg from E″ (°C)	Tg from Tanδ (°C)	Peak of Tanδ
S1G0	66.60	70.85	0.22
S1G1	68.74	70.90	0.21
S1G2	69.12	70.96	0.13
S1G3	69.35	70.65	0.13
S2G0	65.35	70.02	0.26
S2G1	66.71	70.45	0.20
S2G2	67.12	70.10	0.19
S2G3	67.32	70.09	0.18
S3GO	67.50	71.47	0.36
S3G1	68.42	71.68	0.34
S3G2	69.58	71.86	0.32
S3G3	69.89	71.34	0.31
S4G0	68.21	74.00	0.38
S4G1	69.71	74.16	0.29
S4G2	71.21	74.52	0.12
S4G3	68.76	74.19	0.24
S5G0	62.22	67.77	0.46
S5G1	62.25	67.86	0.19
S5G2	63.94	68.10	0.15
S5G3	62.21	67.98	0.19
